# Prospective associations of parental smoking, alcohol use, marital status, maternal satisfaction, and parental and childhood body mass index at 6.5 years with later problematic eating attitudes

**DOI:** 10.1038/nutd.2013.40

**Published:** 2014-01-06

**Authors:** K H Wade, O Skugarevsky, M S Kramer, R Patel, N Bogdanovich, K Vilchuck, N Sergeichick, R Richmond, T Palmer, G Davey Smith, M Gillman, E Oken, R M Martin

**Affiliations:** 1 School of Social and Community Medicine, University of Bristol, Bristol, UK; 2 Medical Research Council (MRC)/University of Bristol Integrative Epidemiology Unit (IEU), University of Bristol, Bristol, UK; 3 Psychiatry and Medical Psychology Department, Belarusian State Medical University, Minsk, Belarus; 4 Department of Pediatrics and Epidemiology, Biostatistics and Occupational Health, McGill University Faculty of Medicine, Montreal, Quebec, Canada; 5 The National Research and Applied Medicine Mother and Child Centre, Minsk, Belarus; 6 Obesity Prevention Program, Department of Population Medicine, Harvard Medical School and Harvard Pilgrim Health Care Institute, Boston, MA, USA; 7 Bristol Biomedical Research Unit in Nutrition, National Institute for Health Research, Bristol, UK

**Keywords:** body mass index, maternal satisfaction, parental smoking and alcohol use, problematic eating attitudes, cohort study, instrumental variable analysis

## Abstract

**Background:**

Few studies have prospectively investigated whether early-life exposures are associated with pre-adolescent eating attitudes.

**Objective:**

The objective of this study is to prospectively investigate associations of parental smoking, alcohol use, marital status, measures of maternal satisfaction, self-reported parental body mass index (BMI) and clinically measured childhood BMI, assessed between birth and 6.5 years, with problematic eating attitudes at 11.5 years.

**Methods:**

Observational cohort analysis nested within the Promotion of Breastfeeding Intervention Trial, a cluster-randomised trial conducted in 31 maternity hospitals and affiliated polyclinics in Belarus. Our primary outcome was a Children's Eating Attitudes Test (ChEAT) score ⩾22.5 (85th percentile), an indicator of problematic eating attitudes. We employed multivariable mixed logistic regression models, which allow inference at the individual level. We also performed instrumental variable (IV) analysis using parents' BMIs as instruments for the child's BMI, to assess whether associations could be explained by residual confounding or reverse causation.

**Subjects:**

Of the 17 046 infants enrolled between 1996 and 1997 across Belarus, 13 751 (80.7%) completed the ChEAT test at 11.5 years.

**Results:**

In fully adjusted models, overweight children at age 6.5 years had a 2.14-fold (95% confidence interval (CI): 1.82, 2.52) increased odds of having ChEAT scores ⩾85th percentile at age 11.5 years, and those who were obese had a 3.89-fold (95% CI: 2.95, 5.14) increased odds compared with normal-weight children. Children of mothers or fathers who were themselves overweight or obese were more likely to score ⩾85th percentile (*P* for trend ⩽0.001). IV analysis was consistent with a child's BMI causally affecting future eating attitudes. There was little evidence that parental smoking, alcohol use, or marital status or maternal satisfaction were associated with eating attitudes.

**Conclusion:**

In our large, prospective cohort in Belarus, both parental and childhood overweight and obesity at 6.5 years were associated with pre-adolescent problematic eating attitudes 5 years later.

## Introduction

Adolescent eating disorders, including anorexia nervosa, bulimia nervosa and binge-eating disorder, affect 0.3–1.6% of boys and girls aged 13–18 years in the USA, with an additional 2.5–10% being affected by subthreshold eating conditions.^1, 2^ However, early diagnosis, treatment and support are expensive, time consuming and infrequently successful.^3^

Parental lifestyle and family environment affect eating attitudes from early childhood.^4, 5^ For example, children exposed to maladaptive parental behaviour and an overprotective family environment are more likely to develop eating disorders.^6, 7, 8^ Problematic environments include those characterised by arguments or physical abuse, and are associated with parental substance and alcohol usage,^9^ smoking^10^ or marital difficulties.^11^ Worries about body image, weight-loss attempts and problematic eating attitudes in early adolescence are associated with a child's own weight, low self-esteem, parental weight, comments about the child's weight and food restriction aimed by mothers towards their children.^7, 12, 13, 14^ Children (particularly girls) of controlling mothers report a restrained eating style that suppresses feelings of hunger, a characteristic associated with overeating, excess weight gain and unhealthy eating behaviours,^15, 16^ and having negative emotions (sadness, guilt or shame) about eating.^17, 18^ These features (overeating, restraint and negative self-esteem) are apparent from childhood to early adulthood.^4, 14, 15, 16, 17, 18, 19, 20, 21, 22, 23^

Studies of family influences on pre-adolescent eating disorders and behaviours have been small (in studies identified by us, the median sample size was 290, the largest comprising 2862 girls^6, 7, 10, 11, 12, 13, 14, 24^); therefore, power may have been insufficient to detect true associations. Most studies have been cross-sectional and are prone to reverse causation (whereby, for example, pre-adolescent body mass index (BMI) could be a consequence, rather than cause, of problematic eating attitudes). In addition, previous studies included information on few covariables and hence were limited in their ability to control for potential confounders. We present prospective associations of early-life exposures with problematic eating attitudes among 13 751 pre-adolescents from the Promotion of Breastfeeding Intervention Trial (PROBIT) in the Republic of Belarus. Situated in Eastern Europe, Belarus is a middle-income, former republic of the USSR (Introduction::Belarus, https://www.cia.gov/library/publications/the-world-factbook/geos/bo.html), with high levels of adult literacy, clean water supply, good sanitation levels and healthcare coverage, long postnatal follow-up and 3 years obligatory maternal leave and low child mortality; however, with recent major economic changes, Belarus has a relatively low Gross Domestic Product and high rates of premature adult mortality.^25^ We investigated parental alcohol intake and smoking, parental BMI, early childhood BMI, change in marital status and measures of maternal satisfaction with marriage, child and motherhood, assessed between birth and 6.5 years of age, with the self-reported eating attitudes of the children 5 years later, at 11.5 years of age.

## Materials and methods

We conducted an observational analysis based on children (and parents) enrolled in PROBIT at birth, who attended follow-up visits throughout infancy and at 6.5 and 11.5 years of age. Trial methods have been described previously.^26^ Briefly, PROBIT was a multicentre, cluster-randomised controlled trial of an intervention to promote increased breastfeeding duration and exclusivity, conducted in the Republic of Belarus. Conducted between June 1996 and December 1997, the trial enrolled 17 046 infants born in 1996–1997 from 31 maternity hospitals randomly assigned to the experimental arm (*n*=16) or control group (usual practices in operation, *n*=15). Inclusion criteria specified that infants were full term (⩾37 weeks gestation), healthy singletons, had a birth weight ⩾2.5 kg and 5 min Apgar score of ⩾5, and that mothers were healthy and initiated breastfeeding. Infants (16 492 (96.7%)) were followed up regularly up to 12 months of age (PROBIT I). Follow-up was administered by principal investigators at the University of Bristol, McGill University, Harvard Medical School, and the Mother and Child Centre, Minsk. The intervention substantially increased breastfeeding duration and exclusivity versus the control arm: at 3 months, intervention infants were seven times more likely to be exclusively (43.3% vs 6.4%) and twice as likely to be predominantly (51.9 vs 28.3%) breastfed, and were breastfed to any degree at higher rates throughout infancy.^26^ Children (13 889 (81.5%)) were followed up at 6.5 years (PROBIT II) and 13 879 (81.4%) were followed up at 11.5 years (PROBIT III), where 13 751 (80.7%) had usable completed Children's Eating Attitudes Test (ChEAT) data (6675 girls and 7076 boys; Supplementary Figure 1).

### Measurement of exposures

A parent or guardian (usually the child's mother) completed questionnaires that elicited information on maternal smoking status and alcohol consumption, at recruitment (relating to exposure during pregnancy) and at 6.5 years. Paternal smoking and alcohol consumption were reported at 6.5 years only. Smoking status was reported as number of cigarettes per day and alcohol intake was reported as frequency (<1 or 1–3 times per month and 1, 2 or >2 times per week) and amount consumed (0–50, 50–150, 150–250, 250–400 or >400 ml) by each parent. Maternal smoking was used as a binary variable (yes/no) and paternal smoking was categorised as 0, 1–9, 10–19 or ⩾20 cigarettes per day. Categorisation for parental smoking was different, as few women smoked large numbers of cigarettes. As in previous studies,^25, 27^ parental alcohol intake was derived by combining responses to questions at each time on the frequency and amount of alcohol consumed, comparing <1 versus ⩾1 unit per week for mothers and <2, 2–4 and >4 units per week for fathers, with 1 unit equal to 25 ml of vodka/other spirits. To capture long-term alcohol and smoking habits among mothers,^28^ we characterised maternal alcohol use and smoking during pregnancy only, postnatal period only, both pre- and post-natal periods, or not at all.

At the child's birth, mothers reported their marital status, classified as registered marriage, unregistered (‘common law') marriage or unmarried. At 6.5 years, their current marital status was reported using the following dichotomous questions: (i) married (registered/unregistered) and living with the same husband as when the child was born, (ii) married (registered) with a different husband, (iii) married (unregistered) with a different husband, (iv) divorced, (v) separated or (vi) widowed. From this, we generated a categorical variable reflecting whether the mother's marital status had changed between birth and at 6.5 years from the child's point of view: (i) married with the same husband (stable two-parent family), (ii) married with a different husband (transition into a step family), (iii) separated/divorced/widowed (transition into a single-parent family) and (iv) stayed unmarried (stable single-parent family). At 6.5 years, mothers reported their level of satisfaction with their child, husband and motherhood on a scale from 1 (very dissatisfied) through 3 (satisfied) to 7 (perfectly satisfied). We collapsed these variables into ‘dissatisfied' (1–3), ‘satisfied' (4–5) and ‘perfectly satisfied' (6–7). Associations relating paternal smoking/alcohol consumption, and the mother's satisfaction with her husband, to ChEAT scores were limited to stable marriages, because it was unclear to which man the mother's response referred in marriages with a new husband or partner.

Weights and heights of the children at 6.5 and 11.5 years were measured by paediatricians at research clinics, as described previously.^29, 30^ At 6.5 years, mothers reported their own and the child's father's weight and height for most (91%) children; a minority (8%) of the fathers/guardians reported for both parents. BMI was calculated as weight (kg) divided by height (m^2^). As in previous papers,^25, 27, 30^ childhood thinness, overweight and obesity were defined by age- and sex-specific models recommended by Cole *et al.*,^31, 32^ with trajectories equivalent at age 18 years to the World Health Organization's defined BMI thresholds of ⩽17 kg m^−2^ (thinness), >17 to <25 kg m^−2^ (normal weight), ⩽25 to ⩽30 kg m^−2^ (overweight) and ⩾30 kg m^−2^ (obesity).^33^ Parental BMI was categorised according to these WHO thresholds.^33^ Assumed to be errors, height and weight measurements in excess of ±4 s.d. from the mean (29 mothers, 27 fathers and 125 children) were excluded from analyses.

### Measurement of eating attitudes

At the PROBIT III research clinic, children self-completed a modified version of ChEAT, originally a 26-item questionnaire assessing a variety of eating attitudes and behaviours on a Likert scale, ranging from 1 (always) to 6 (never).^34^ Children were asked to complete the questionnaire without interference from either parents or the paediatricians. Each question contributes to an assessment of problematic eating attitudes, including food preoccupation, peer and media pressure about eating, weight and body image, dieting, purging and restriction of food. ChEAT is therefore a quantitative indicator of problematic eating attitudes that may be symptomatic of more severe eating disorders. One study based on the UK general practice found that 10% of young adults who scored highly on the related Eating Attitudes Test had a clinical diagnosis of disordered eating, and more than one-third had clinically important concerns and weight preoccupation^35^ compared with randomly selected individuals who scored below the threshold, where no full or partial disordered eating syndromes were found. In addition, recent studies have shown that ChEAT is positively correlated with other validated measures of disturbed eating, including the Eating Disorder Examination Adapted for Children, Revised Eating Disorder Inventory-Body Dissatisfaction Subscale, Rosenburg Self Esteem Scale and Child Depression Inventory.^36^ We translated the ChEAT into Russian and then back to English to verify meaning; we are not aware of any validation studies in Belarus or other Russian-speaking populations.

As Maloney *et al.*^37^ found that one question (‘I can show self-control around food') was negatively correlated with the questionnaire, we administered 25 questions only, where the original six-item Likert scale was simplified to a three-item scale: ‘often', ‘sometimes' and ‘never'. In preliminary factor analyses, question 25 (‘I enjoy trying new rich foods') was inversely correlated with total ChEAT score and was removed from the analysis, which was based on the 24-item ChEAT-26. Responses were scored as 3 (‘often'), 1.5 (‘sometimes') and 0 (‘never'), giving a 0–72 range for ChEAT-24, a similar range (0–78) from ChEAT-26.

In previous work within Caucasian populations from Europe, North America and Australia, children with high ChEAT scores, ranging between the 75th and 91st percentiles, were predisposed to eating disorders.^22, 34, 36, 37, 38, 39^ Moreover, lower thresholds can generate more false-positive results than higher thresholds.^36, 40^ Therefore, we defined our threshold closer to the upper end of the range for problematic eating attitudes as a ChEAT-24 score ⩾22.5, corresponding to the 85th percentile in our data. In a sensitivity analysis, we investigated associations using a ChEAT-24 score ⩾25.5 (91st percentile, comparable to Maloney *et al.*^37^). We did not investigate ChEAT-24 scores as a continuous outcome, as the scores were positively skewed and had a bimodal distribution (10.1% of children reported total scores of 0, that is, answered ‘never' to all 24 questions).

An audit was conducted an average of 1.3 years (range 0.2–2.4) after the initial clinic visit to assess the reproducibility of the polyclinic data. Percentage agreement for ChEAT scores ⩾85th percentile was 85.1% comparing original and audit results for 141 randomly selected children with complete ChEAT scores for both visits. Cohen's kappa for chance-corrected agreement was 0.46 (95% confidence interval (CI): 0.30, 0.63), indicating moderate test–retest reproducibility.

### Ethics

PROBIT III was approved by the Belarusian Ministry of Health and received ethical approval from the McGill University Health Centre Research Ethics Board, the Human Subjects Committee at Harvard Pilgrim Health Care and the Avon Longitudinal Study of Parents and Children Law and Ethics Committee. A parent/legal guardian provided written informed consent in Russian at enrolment and at follow-up visits, and all children provided written assent at 11.5 years.

### Statistical analysis

To assess how each question contributed to the variance of ChEAT scores, we conducted a principal components analysis to verify the factor structure of the ChEAT questionnaire (Supplementary Table 1). Factors obtained in our analyses were similar to those reported previously.^34^ We investigated associations of exposures measured during infancy and at 6.5 years with problematic eating attitudes (ChEAT score ⩾85th percentile) at 11.5 years using multivariable mixed logistic regression models; these employed the ‘xtmelogit' command in STATA (STATA Corp, College Station, TX, USA), which allows inference at the individual level within clusters (there was a moderate degree of within-polyclinic clustering of ChEAT responses; intraclass correlation coefficient=0.22). We built the following cluster-adjusted models: a basic model controlling for age and sex, and a fully adjusted model additionally controlling for location of polyclinic (urban/rural and East/West Belarus), treatment group (intervention/control), parental education measured at PROBIT I (initial, incomplete or common secondary, advanced secondary or partial university, and completed university) and highest household occupation (manual worker (including farmer)/service worker (non-manual) categorised as in previous studies^25, 27^). We adjusted for treatment group, because in an intention-to-treat analysis comparing the children randomly allocated to the experimental intervention versus control groups, the proportion of 11.5-year-old children with problematic eating attitudes was significantly lower in the promotion of breastfeeding arm (Skugarevsky *et al.* Psychiatry and Medical Psychology Department, Belarusian State Medical University, unpublished manuscript). As associations between parental smoking and alcohol intake, marital status and maternal satisfaction variables and ChEAT scores did not differ between models, we present fully adjusted models only. We additionally adjusted for maternal smoking when analysing associations of parental and childhood BMI with ChEAT-24 scores, as maternal smoking is associated with childhood obesity.^41^

We compared ChEAT scores per s.d. increase in parental and child BMI. As few fathers were underweight (*n*= 5), these men were excluded. We present odds ratios (ORs), 95% CIs and *P*-values giving the odds of having a ChEAT-24 score ⩾85th percentile, comparing the highest versus lowest (reference) levels of a binary variable, per-unit increase in an ordered categorical variable or per s.d. increase in BMI. For defined categories of BMI, normal weight in children, mothers and fathers was considered as the reference category, with which the underweight, overweight and obese categories were compared. We found no evidence of sex interaction (*P*-values for interaction were >0.23) with exposures, except the mother's satisfaction with her child (*P-*value for interaction=0.01); thus, we present associations for girls and boys combined.

Within one polyclinic site (‘L' *n*=928), 76% of the respondents answered ‘never' to all ChEAT questions and 2.3% scored ⩾22.5. Therefore, we performed a sensitivity analysis excluding polyclinic site ‘L' to determine its effect on results.

Finally, we performed instrumental variable (IV) analysis using the parents' BMIs as instruments for the child's BMI, to assess whether prospective associations of the child's BMI with problematic eating attitudes were causal and not explained by residual confounding or reverse causation.^42^ An IV is reliably associated with a risk factor (here, the child's BMI) and with the outcome (here, problematic eating attitudes) only because of its association with the risk factor.^43^ The instrument must not be associated with confounding factors and must not be influenced by the outcome so as not to be biased by reverse causation (see Table 1 of Davey Smith *et al.*^42^). We used the BMI of each parent as the ‘instrument' because parents' BMIs are positively associated with the child's BMI^42^ (Pearson's correlation coefficient=0.2 in PROBIT); associations of potential confounders with parents' BMIs were weaker than child BMI and, for some, in the opposite direction;^27^ and unless problematic eating attitudes in the child act as a strong marker for their parents' eating attitudes, they are unlikely to cause variation in parental BMI, avoiding reverse causation. In an IV analysis, the component of variation within the risk factor of interest explained by the instrument is used to provide an unbiased and unconfounded assessment of causality between the risk factor and outcome. We used the ‘gmm' command in STATA to compute IV estimates using the BMI of the mother and father as separate instruments for their child's BMI. We compared ORs per s.d. increase in child's BMI obtained from conventional linear regression and IV analysis.

We conducted all analyses using STATA version 12 (STATA Corp).

## Results

Of the 17 046 children enrolled, 13 879 (81.4%) attended the PROBIT III visit at a median age of 11.5 years (interquartile range, 11.3–11.8). Of these, 13 751 (80.7%) had usable and complete ChEAT data and 48.5% were girls (Table 1). At 6.5 years, 2.1% of children (223 girls and 67 boys) were underweight, 7.2% (498 girls and 495 boys) were overweight and 2.0% (148 girls and 127 boys) were obese; 10.4% and 8.2% of mothers and fathers, respectively, were obese.

Girls were more likely to have ChEAT scores ⩾85th percentile (20.8% vs 14.1%, respectively) than boys. No other potential confounders were associated with ChEAT scores ⩾85th percentile, apart from an inverse association with age (Table 2). There were no important associations of parental smoking or alcohol intake, change in marital status or maternal satisfaction with problematic eating attitudes (Table 3). In the fully adjusted model, the child's BMI measured at 6.5 years was positively associated with problematic eating at 11.5 years: 1 s.d. increase in BMI was associated with a 34% increased odds (95% CI: 29%, 40%) of having ChEAT scores ⩾85th percentile (Table 4). Both maternal and paternal BMI at 6.5 years were positively associated with problematic eating attitudes at 11.5 years (fully adjusted OR per s.d. increase in BMI: 1.09 (95% CI: 1.04, 1.15) and 1.09 (95% CI: 1.03, 1.14) for mothers and fathers, respectively).

Compared with normal-weight children, overweight children at age 6.5 years had >2-fold increased odds of having ChEAT scores ⩾85th percentile at age 11.5 years (fully adjusted OR: 2.14; 95% CI: 1.82, 2.52), and those who were obese, nearly a 4-fold increased odds (fully adjusted OR: 3.89; 95% CI: 2.95, 5.14) (Table 4). In addition, children of mothers or fathers who were themselves overweight or obese were more likely to score ⩾85th percentile on the ChEAT questionnaire (*P* for trend ⩽0.001).

Results were similar in the sensitivity analysis excluding polyclinic site ‘L' and when using the stricter threshold of the 91st percentile (Supplementary Tables 2 and 3). In IV analyses, the child's BMI was positively associated with ChEAT scores ⩾85th percentile (OR per s.d. increase in the child's BMI: 1.46 (95% CI: 1.28, 1.67) and 1.42 (95% CI: 1.22, 1.65), using maternal and paternal BMI as instruments, respectively; Supplementary Table 4). These estimates were similar to the effect estimate obtained from conventional linear regression model.

## Discussion

In our large, prospective cohort study in Belarus, both parental BMI and childhood BMI at 6.5 years were positively associated with pre-adolescent problematic eating attitudes 5 years later. There was little evidence that parental smoking, maternal alcohol intake, change in marital status or maternal satisfaction measures were associated with future problematic eating attitudes among the offspring.

The observation that children with higher BMI at 6.5 years had higher ChEAT scores is consistent with cross-sectional data showing that overweight (BMI ⩾95th percentile) and at-risk for overweight (BMI between 85th and 95th percentile) children had higher total ChEAT scores compared with non-overweight children.^44^ In PROBIT, children who were overweight or obese at age 6.5 years had, respectively, twofold and nearly fourfold increased risks of having ChEAT scores ⩾85th percentile 5 years later. Given that nearly 10% of children were overweight or obese at 6.5 years, these results highlight the importance of childhood overweight and obesity for the future development of problematic eating attitudes in later life.

Our results also indicate that the association of BMI with subsequent problematic eating attitudes was equally strong in boys and girls, a finding at odds with some literature, which suggests that being overweight in early life has a stronger influence on developing problematic eating attitudes in girls.^5, 10, 12, 22, 38^ Previously reported parental risk factors for childhood eating disorders include obesity, overprotection, family conflict, eating disorders, imposition of food restriction onto the child and concerns about the parents own or their child's weight.^4, 5, 6, 7, 8, 9, 10, 11, 12, 14, 24, 44, 45, 46, 47^ However, as described in the introduction, most previous studies were cross-sectional and had limited power^6, 7, 10, 11, 12, 13, 14, 24^. In agreement with prospective studies that used ChEAT, girls had a greater risk of problematic eating attitudes than boys.^22, 38^

Children of overweight parents are at greater risk of becoming overweight themselves, as body weight and shape have strong genetic and family environmental determinants.^48, 49, 50^ Overweight children as young as 5 years old report lower body esteem than thinner children, perhaps because thinner body shapes are supported by social and parental pressures^51^ in contrast to heavier body weights.^5^ Being overweight as a child or having overweight parents may contribute to the development of problematic eating attitudes as an approach to controlling weight.

Our study strengths are its large sample size generating precise effect estimates, excellent follow-up rate and prospective data collection, but there are limitations. As the parental weights and heights were verbally reported, they may be prone to measurement error and reporting bias. However, as parental information was reported years before ChEAT scores were collected, any error should be random, attenuating rather than inflating observed associations. Other potential confounders, including physical activity of the child, presence of parental eating disorders, cultural perceptions of body image^47^ and physical/sexual abuse,^8^ were not measured. Within this cohort, the prevalence of overweight and obesity among children and mothers was considerably lower compared with many other countries, particularly in the United States.^52^ By extension, these findings may therefore be of greater importance in areas where the burden of childhood obesity is a much greater national health problem. On the other hand, due to varying socioeconomic and confounding structures between countries, these results may not generalise to areas with different levels of overweight and obesity.

Although BMI was measured 5 years before problematic eating assessment, it is possible that problematic eating attitudes were already present by 6.5 years;^8^ however, information on eating attitudes was not available before 11.5 years. Thus, any observed association of BMI at 6.5 years with eating attitudes at 11.5 years could reflect an influence of pre-existing disordered eating on BMI. However, there are two pieces of evidence supporting the inference that a child's BMI causally affects future eating attitudes. First, IV and conventional regression estimates were similar. Although there may be some residual bias, our IV analyses using parents' BMI as a proxy for the child's BMI should be less affected by confounding or reverse causation than the conventional regression analyses. However, as eating disorders may be partly heritable (evidence suggests that ∼54% of the variance in 11-year-old pubertal and 17-year-old twins is explained by genetic factors^53^), and as there is a possible genetic correlation between disordered eating and BMI,^8^ we cannot completely disregard reverse causation.

Second, in intention-to-treat analyses comparing children randomly allocated to the intervention versus control arm of PROBIT (Skugarevsky, *et al.* Psychiatry and Medical Psychology Department, Belarusian State Medical University, unpublished manuscript), the intervention was associated with a reduced risk of problematic eating attitudes at 11.5 years; however, the intervention had no effect on BMI at 6.5^29^ or 11.5 years.^30^ If eating attitudes influenced child's BMI, the breastfeeding promotion intervention would have an effect on both BMI and eating attitudes, which we do not observe.

The predictive value of the ChEAT questionnaire in relation to future risk of developing an eating disorder is uncertain. Nevertheless, studies using alternative measures of disordered eating have shown that children expressing problematic eating attitudes in early life have an increased risk of developing eating disorders.^46, 54^ For example, children aged 1–10 years with eating conflicts and struggles with food, as assessed by maternal interview, had a six- to sevenfold increased risk of being diagnosed with anorexia nervosa in adolescence and young adulthood.^54^ However, it is unclear how other measures of problematic eating attitudes can approximate the prognostic value of the ChEAT questionnaire, or what the optimum threshold for defining problematic eating attitudes should be.^19, 34, 37, 38, 55, 56^ In our study, the use of either the 85th or 91st percentiles as thresholds for problematic eating gave similar results.

In conclusion, our study showed that parental and childhood BMI at 6.5 years were positively associated with problematic eating attitudes 5 years later. We observed little evidence of associations of parental smoking and alcohol intake, change in marital status, or maternal satisfaction measures with offspring problematic eating attitudes. Our results highlight the potential public health importance of preventing childhood overweight in early primary school years for future avoidance of disturbed eating attitudes and behaviours.

## Figures and Tables

**Table 1:** Characteristics of participants in the PROBIT, Belarus, followed up at age 11.5 years

*Characteristics*	*Total number of individuals for each variable (N)*	*Percentages (%) or mean (s.d.) or median (IQR) where specified (*N*=13 751)*
Female (%)	13 751	48.5
Median (IQR) age at physical examination (years)	13 730	11.5 (11.3–11.8)
Urban vs rural (% in urban)	13 751	57.9
West vs East of Belarus (% in west)	13 751	52.6
Within intervention arm (%)	13 751	53.5
*Mother's alcohol intake from pregnancy to PROBIT II (*N*=12 531)*^a^		
Consumed <1 unit per week	5412	43.2
Prenatal only	88	0.7
Postnatal only	6721	43.6
Pre- and post-natal	310	2.5
*Mother's smoking from pregnancy to PROBIT II (*N*=12 339)*		
None	10 517	85.2
Prenatal only	81	0.7
Postnatal only	1537	12.5
Pre- and postnatal	204	1.7
Father's smoking at 6.5 years (% in highest category)^b^	10 266	7.4
Father's alcohol intake at 6.5 years (% in highest category)^b^	10 243	27.1
Marital status (% persistent marriage)	12 532	82.4
Mother's satisfaction with husband (% perfectly satisfied) at 6.5 years	10 176	32.6
Mother's satisfaction with child (% perfectly satisfied) at 6.5 years	12 575	63.5
Mother's satisfaction with motherhood (% perfectly satisfied) at 6.5 years	12 539	78.0
*Maternal BMI category (%) at PROBIT II (*N*=12 719)*^c^		
Underweight	75	0.6
Normal	7949	62.5
Overweight	3265	25.7
Obese	1430	11.2
*Paternal BMI category (%) at PROBIT II (*N*=11 609)*^c^		
Underweight	5	0.04
Normal	5461	47.0
Overweight	5010	43.2
Obese	1133	9.8
*Child BMI category (%) at PROBIT II (*N*=12 851)*^c^		
Underweight	290	2.3
Normal	11 293	87.9
Overweight	993	7.7
Obese	275	2.1
*Child BMI category (%) at PROBIT III (*N*=13 741)*^c^		
Underweight	338	2.5
Normal	11 283	82.1
Overweight	1732	12.6
Obese	388	2.8
Highest household occupation (% non-manual workers)	12 836	56.6
Mother's education (% completed university)	13 751	13.6
Father's education (% completed university)	13 316	13.2
ChEAT scores ⩾85th percentile (that is, score ⩾22.5)	13 751	17.3

Abbreviations: BMI, body mass index; ChEAT, Children's Eating Attitudes Test;^34^ IQR, interquartile range; PROBIT, Promotion of Breastfeeding Intervention Trial; WHO, World Health Organization.

a Mother's alcohol consumption ⩾1 unit per week by period or father's alcohol consumption >4 units per week.

b Highest category for father's smoking was ⩾20 cigarettes per day.

c Categories of BMI for underweight, overweight and obesity in children were defined by Cole *et al.*^31, 32^ and are mapped onto the WHO categories for adults. The WHO definitions were used for adults.

**Table 2:** Association between potential confounders and ChEAT scores ⩾85th percentile in PROBIT, Belarus

*Confounders*^a^	N *(%) of ChEAT scores* ⩾*22.5*	*OR (95% CI)*^b^	P*-value*
Urban (city) (*n*=7956)	1300 (16.3)	1.00 (Ref)	
Rural (village) (*n*=5795)	1082 (18.7)	1.08 (0.51, 2.29)	0.84
West of Belarus (*n*=7238)	1165 (16.1)	1.00 (ref)	
East of Belarus (*n*=6513)	1217 (18.7)	1.27 (0.60, 2.69)	0.53
*Age of child (years)*^c^			
10.2–11.4 (*n*=4583)	844 (18.4)		
11.4–11.7 (*n*=4589)	774 (16.9)		
11.7–14.5 (*n*=4558)	758 (16.6)	0.90 (0.84, 0.96)	0.001^d^
*Sex (female vs male)*^e^			
Male (*n*=7076)	997 (14.1)	1.00 (ref)	
Female (*n*=6675)	1385 (20.8)	1.62 (1.47, 1.77)	<0.0001
*Maternal education*			
Incomplete secondary or common (*n*=4823)	865 (17.9)		
Advanced secondary or partial (*n*=7064)	1216 (17.2)		
Completed university (*n*=1864)	301 (16.2)	1.00 (0.93, 1.08)	0.97^d^
*Paternal education*			
Incomplete secondary or common secondary (*n*=5232)	895 (17.1)		
Advanced secondary or partial university (*n*=6328)	1127 (17.8)		
Completed university (*n*=1756)	285 (16.2)	1.04 (0.97, 1.12)	0.26^d^
*Highest household occupation*			
Manual worker/farmer (*n*=5571)	995 (17.9)	1.00 (ref)	
Non-manual worker (*n*=7265)	1237 (17.0)	0.99 (0.89, 1.09)	0.80

Abbreviations: ChEAT, Children's Eating Attitudes Test;^34^ CI, confidence interval; OR, odds ratio; PROBIT, Promotion of Breastfeeding Intervention Trial; Ref, reference group.

a *N*-values in each row heading represent the number of individuals within each cell of that row, respectively, where percentages within each cell are proportions of the corresponding *N*-value. For example, 18.7% (1082/5 795) of individuals who live in rural areas have ChEAT scores ⩾22.5 compared with 16.3% (1300/7956) of individuals who live in urban areas.

b All effect-estimates account for age, sex and clustering by hospital/polyclinic and represent the OR, giving the change in odds (95% CI) of having a ChEAT score ⩾22.5 (85th percentile) per change or unit increase in the level of each binary or ordered categorical variable, respectively. For example, an OR of 1.08 (95% CI: 0.51, 2.29; *P*=0.84) indicates that there is a 8% increased odds of having a ChEAT score above the 85th percentile among individuals who live in rural areas compared with those who live in urban areas.

c Not adjusted for age.

d OR and *P*-value for trend.

e Not adjusted for sex.

**Table 3:** Associations of family exposures with problematic eating attitudes at age 11.5 years (ChEAT scores ⩾85th percentile) in PROBIT, Belarus

*Exposures*	*% of ChEAT scores* ⩾*85th percentile*^a^	*Fully adjusted OR*^b^	*95% CI*
*Maternal smoking from pregnancy to PROBIT II*			
None (*n*=10 517)	17.3	1.00 (Ref)	
Prenatal only (*n*=81)	12.4	0.76	0.35, 1.69
Postnatal only (*n*=1537)	20.8	1.12	0.96, 1.31
Both pre- and postnatal (*n*=204)	21.6	0.97	0.63, 1.48
*P*-value for heterogeneity		0.51	
*Maternal alcohol intake from pregnancy to PROBIT II*			
<1 Unit per week throughout (*n*=5412)	17.5	1.00 (Ref)	
⩾1 Unit per week prenatal only (*n*=88)	18.2	1.13	0.61, 2.11
⩾1 Unit per week postnatal only (*n*=6721)	18.2	1.11	0.99, 1.24
⩾1 Unit per week pre- and postnatal (*n*=310)	10.0	1.00	0.62, 1.62
*P*-value for heterogeneity		0.91	
*Paternal smoking at PROBIT II (cigarettes per day)*			
None (*n*=3791)	17.6	1.00 (Ref)	
1–9 (*n*=3084)	18.1	1.00	0.88, 1.15
10–19 (*n*=2635)	18.2	1.05	0.91, 1.20
⩾ 20 (*n*=756)	19.6	1.05	0.84, 1.30
OR per smoking category		1.02	0.96, 1.08
*P*-value for trend		0.50	
*Paternal alcohol intake at PROBIT II*			
<2 Units per week (*n*=4656)	18.3	1.00 (Ref)	
2–4 Units per week (*n*=2815)	16.9	0.98	0.85, 1.12
>4 Units per week (*n*=2772)	18.8	1.08	0.94, 1.24
OR per alcohol intake category		1.03	0.97, 1.11
*P*-value for trend		0.33	
*Marital status at birth and after 6.5 years*			
Married, same husband (*n*=10 329)	18.0	1.00 (ref)	
Married, different husband (*n*=617)	20.1	1.04	0.82, 1.33
Separated/divorced/widowed (*n*=1373)	17.1	0.97	0.82, 1.15
Stayed unmarried (*n*=213)	13.2	0.88	0.35, 2.18
*P*-value for heterogeneity		0.88	
*Marital satisfaction*			
Dissatisfied (*n*=953)	17.5	1.00 (ref)	
Satisfied (*n*=5908)	18.0	1.05	0.87, 1.28
Perfectly satisfied (*n*=3315)	18.2	1.00	0.81, 1.23
OR per satisfaction category		0.98	0.90, 1.08
*P*-value for trend		0.70	
*Child satisfaction*			
Dissatisfied (*n*=213)	16.9	1.00 (ref)	
Satisfied (*n*=4377)	17.3	0.81	0.54, .122
Perfectly satisfied (*n*=7985)	18.3	0.73	0.49, 1.10
OR per satisfaction category		0.89	0.81, 0.99
*P*-value for trend		0.03	
*Motherhood satisfaction*			
Dissatisfied (*n*=85)	18.8	1.00 (ref)	
Satisfied (*n*=2675)	18.0	1.12	0.60, 2.11
Perfectly satisfied (*n*=9779)	17.9	1.02	0.55, 1.92
OR per satisfaction category		0.93	0.82, 1.05
*P*-value for trend		0.22	

Abbreviations: ChEAT, Children's Eating Attitudes Test;^34^ CI, confidence interval; OR, odds ratio; PROBIT, Promotion of Breastfeeding Intervention Trial; Ref, reference group.

a ORs (and *P*-value) for trend represent the change in odds (95% CI) of having ChEAT scores ⩾22.5 (85th percentile) per-unit increase in each ordered categorical variable.

b Adjusted for age, sex, location of polyclinic, treatment group, maternal/paternal occupation/education and polyclinic site.

**Table 4:** Associations of child's and parents' BMI at Age 6.5 years with problematic eating attitudes at age 11.5 years (ChEAT scores ⩾85th percentile) in PROBIT, Belarus

*Exposures*^a^	*% ChEAT scores* ⩾*22.5*	*Basic OR*^b^	*95% CI*	*Adjusted OR*^c^	*95% CI*
*Child's BMI, PROBIT II (kg m^−2^)*					
Normal (*n*=11293)	16.5	1.00 (Ref)		1.00 (Ref)	
Underweight (*n*=290)	15.5	0.87	0.62, 1.22	0.84	0.59, 1.20
Overweight (*n*=993)	28.5	2.16	1.85, 2.53	2.14	1.82, 2.52
Obese (*n*= 275)	40.4	3.80	2.90, 4.97	3.89	2.95, 5.14
OR per s.d.		1.33	1.28, 1.39	1.34	1.29, 1.40
*P*-value for trend		<0.0001		<0.0001	
*Maternal BMI, PROBIT II (kg m^−2^)*					
Normal (*n*=7949)	17.2	1.00 (Ref)		1.00 (Ref)	
Underweight (*n*=75)	17.3	0.97	0.52, 1.80	1.18	0.63, 2.23
Overweight (*n*=3265)	18.3	1.10	0.98, 1.23	1.10	0.98, 1.23
Obese (*n*=1430)	20.8	1.29	1.11, 1.49	1.29	1.11, 1.50
OR per s.d.		1.09	1.05, 1.14	1.09	1.04, 1.15
*P*-value for trend		0.0001		0.0002	
*Paternal BMI at PROBIT II (kg m^−2^)*					
Normal (*n*=5461)	17.2	1.00 (Ref)		1.00 (Ref)	
Overweight (*n*=5010)	18.4	1.09	0.98, 1.22	1.11	0.99, 1.24
Obese (*n*=1133)	20.5	1.28	1.08, 1.51	1.27	1.06, 1.51
OR per s.d.		1.08	1.03, 1.13	1.09	1.03, 1.14
*P*-value for trend		0.001		0.001	

Abbreviations: BMI, body mass index (kg m^−2^); ChEAT, Children's Eating Attitudes Test;^34^ CI, confidence interval; OR, odds ratio; PROBIT, Promotion of Breastfeeding Intervention Trial; Ref, reference group; WHO, World Health Organization.

a Categories of BMI for underweight, overweight and obesity in children were defined by Cole *et al.*^31, 32^ and are mapped onto the WHO categories for adults. The WHO definitions were used for adults.

b All effect-estimates are adjusted for age, sex and clustering by hospital/polyclinic and represent the OR giving the change in odds (95% CI) of having a ChEAT score ⩾22.5 (85th percentile) per s.d. increase in BMI (kg m^−2^).

c Adjusted for age, sex, location of polyclinic, treatment group, maternal/paternal occupation/education, maternal smoking status from pregnancy to PROBIT II and cluster (polyclinic site).

## References

[bib1] Swanson SA, Crow SJ, Le Grange D, Swendsen J, Merikangas KR. Prevalence and correlates of eating disorders in adolescents. Arch Gen Psychiatry 2011; 68: 714–723. 21383252 10.1001/archgenpsychiatry.2011.22 PMC5546800

[bib2] Jones JM, Bennett S, Olmsted MP, Lawson ML, Rodin G. Disordered eating attitudes and behaviour in teenaged girls: a school-based study. CMAJ 2001; 165: 547–552. 11563206 PMC81412

[bib3] Hoek HW, van Hoeken D. Review of the prevalence and incidence of eating disorders. Int J Eat Disord 2003; 34: 383–396. 14566926 10.1002/eat.10222

[bib4] Savage JS, Fisher JO, Birch LL. Parental influence on eating behavior: conception to adolescence. J Law Med Ethics 2007; 35: 22–34. 17341215 10.1111/j.1748-720X.2007.00111.x PMC2531152

[bib5] Fairburn CG, Brownell KD. Eating Disorders and Obesity: A Comprehensive Handbook 2nd edn The Guilford Press: Oxford, UK, 2002.

[bib6] Galloway AT, Fiorito L, Lee Y, Birch LL. Parental pressure, dietary patterns and weight status among girls who are ‘picky eaters'. J Am Diet Assoc 2005; 105: 451–548. 10.1016/j.jada.2005.01.029 PMC2530930 15800554

[bib7] Smolak L, Levine MP, Schermer F. Parental input and weight concerns among elementary school children. Int J Eat Disord 1999; 25: 263–271. 10191990 10.1002/(sici)1098-108x(199904)25:3<263::aid-eat3>3.0.co;2-v

[bib8] Polivy J, Herman CP. Causes of eating disorders. Annu Rev Psychol 2002; 53: 187–213. 11752484 10.1146/annurev.psych.53.100901.135103

[bib9] Chandy JM, Harris L, Blum RW, Resnick MD. Disordered eating among adolescents whose parents misuse alcohol: protective and risk factors. Int J Addict 1994; 29: 505–516. 8188443 10.3109/10826089409047396

[bib10] Johnson JE, Cohen P, Kasen S, Brook JS. Childhood adversities associated with risk for eating disorders or weight problems during adolescence or early adulthood. Am J Psychiatry 2002; 159: 394–400. 11870002 10.1176/appi.ajp.159.3.394

[bib11] Martínez-González MA, Gual P, Lahortiga F, Alonso Y, de Irala-Estévez J, Cervera S. Parental factors, mass media influences and the onset of eating disorders in prospective population-based cohort. Pediatrics 2003; 111: 315–320. 12563057 10.1542/peds.111.2.315

[bib12] Allen K, Byrne SM, Forbes D, Oddy WH. Risk factors for full- and partial-syndrome early adolescent eating disorders: a population-based pregnancy cohort study. J Am Acad Child Adolesc Psychiatry 2009; 48: 800–809. 19564799 10.1097/CHI.0b013e3181a8136d

[bib13] Jacobi C, Agras WS, Hammer L. Predicting children's reported eating disturbances at 8 years of age. J Am Acad Child Adolesc Psychiatr 2001; 40: 364–372. 10.1097/00004583-200103000-00017 11288779

[bib14] Birch LL, Fisher JO. Mothers' child-feeding practices influence daughters' eating and weight. Am J Clin Nutr 2000; 71: 1054–1061. 10799366 10.1093/ajcn/71.5.1054 PMC2530928

[bib15] Carper JL, Orient Fisher J, Birch LL. Young girls' emerging dietary restraint and disinhibition are related to parental control in child feeding. Appetite 2000; 35: 121–129. 10986105 10.1006/appe.2000.0343

[bib16] van Strien T, Bazelier FG. Perceived parental control of food intake is related to external, restrained and emotional eating in 7-12-year-old boys and girls. Appetite 2007; 49: 618–625. 17512089 10.1016/j.appet.2007.03.227

[bib17] Braet C, Van Strien T. Assessment of emotional, externally induced and restrained eating behaviour in nine to twelve-year-old obese and non-obese children. Behav Res Ther 1997; 35: 863–873. 9299807 10.1016/s0005-7967(97)00045-4

[bib18] Fisher JO, Birch LL. Parents' restrictive feeding practices are associated with young girls' negative self-evaluation of eating. J Am Diet Assoc 2000; 100: 1341–1346. 11103656 10.1016/S0002-8223(00)00378-3 PMC2548290

[bib19] McVey G, Tweed S, Blackmore E. Dieting among preadolescent and young adolescent females. CMAJ 2004; 170: 1559–1561. 15136549 10.1503/cmaj.1031247 PMC400720

[bib20] Knez R, Munjas R, Petrovecki M, Paucic-Kirincic E, Persic M. Disordered eating attitudes among elementary school population. J Adolesc Health 2006; 38: 628–630. 16635782 10.1016/j.jadohealth.2005.04.011

[bib21] Maloney MJ, McGuire J, Daniels SR, Specker B. Dieting behaviour and eating attitudes in children. Pediatrics 1989; 84: 482–489. 2788865

[bib22] Rolland K, Farnill D, Griffiths RA. Body figure perceptions and eating attitudes among Australian schoolchildren aged 8 to 12 years. Int J Eat Disord 1997; 21: 273–278. 9097200 10.1002/(sici)1098-108x(199704)21:3<273::aid-eat7>3.0.co;2-h

[bib23] Birch LL, Fisher JO. Development of eating behaviors among children and adolescents. Pediatrics 1998; 101: 539–549. 12224660

[bib24] Rodriguez MA, Novalbos Ruiz JP, Martínez Nieto JM, Escobar Jiménez L, Castro De Haro AL. Epidemiological study of the influence of family and socioeconomic status in disorders of eating behaviour. Eur J Clin Nutr 2004; 58: 846–852. 15164104 10.1038/sj.ejcn.1601884

[bib25] Patel R, Lawlor DA, Kramer MS, Davey Smith G, Bogdanovich N, Matush L et al. Socioeconomic inequalities in height, leg length and trunk length among children aged 6.5 years and their parents from the Republic of Belarus: Evidence from the Promotion of Breastfeeding Intervention Trial (PROBIT). Ann Hum Bioly 2011; 38: 592–602. 10.3109/03014460.2011.577752 PMC3719034 21591995

[bib26] Kramer MS, Chalmers B, Hodnett ED, Sevkovskaya Z, Dzikovich I, Shapiro S et al. Promotion of Breastfeeding Intervention Trial (PROBIT): a randomized controlled trial in the Republic of Belarus. JAMA 2001; 285: 413–420. 11242425 10.1001/jama.285.4.413

[bib27] Patel R, Lawlor DA, Kramer MS, Davey Smith G, Bogdanovich N, Matush L et al. Socio-economic position and adiposity among children and their parents in the Republic of Belarus. Eur J Public Health 2010; 21: 158–165. 20418336 10.1093/eurpub/ckq041 PMC3451194

[bib28] Yang S DA, Kramer MS. Exposure to parental smoking and child growth and development: a cohort study. BMC Pediatrics 2013; 13: 104. 23842036 10.1186/1471-2431-13-104 PMC3717101

[bib29] Kramer MS, Matush L, Vanilovich I, Platt RW, Bogdanovich N, Sevkovskaya Z et al. Effects of prolonged and exclusive breastfeeding on child height, weight, adiposity, and blood pressure at age 6.5 y: evidence from a large randomized trial. Am J Clin Nutr 2007; 86: 1717–1721. 18065591 10.1093/ajcn/86.5.1717

[bib30] Martin RM, Patel R, Kramer MS, Guthrie L, Vilchuck K, Bogdanovich N et al. Effects of promoting longer-term and exclusive breastfeeding on adiposity and insulin-like growth factor-1 at age 11.5 years: a randomized trial. JAMA 2013; 320: 1240–1243. 10.1001/jama.2013.167 PMC3752893 23483175

[bib31] Cole TJ, Bellizzi MC, Flegal KM, Dietz WH. Establishing a standard definition for child overweight and obesity worldwide: international survey. BMJ 2000; 320: 1240–1243. 10797032 10.1136/bmj.320.7244.1240 PMC27365

[bib32] Cole TJ, Flegal KM, Nicholls D, Jackson AA. Body mass index cut offs to define thinness in children and adolescents: international survey. Br Med J 2007; 335: 194–197. 17591624 10.1136/bmj.39238.399444.55 PMC1934447

[bib33] WHO. Physical Status: The Use and Interpretation of Anthropometry. WHO: Geneva, 1995.

[bib34] Smolak L, Levine MP. Psychometric properties of the Children's Eating Attitudes Test. Int J Eat Disord 1994; 16: 275–282. 7833961 10.1002/1098-108x(199411)16:3<275::aid-eat2260160308>3.0.co;2-u

[bib35] King MB. Eating disorders in a general practice population. Prevalence, characteristics and follow-up at 12 to 18 months. Psychol Med 1989; 14: 1–34. 10.1017/s0264180100000515 2788294

[bib36] Erickson SJ, Gerstle M. Developmental considerations in measuring children's disordered eating attitudes and behaviours. Eat Behav 2005; 8: 224–235. 10.1016/j.eatbeh.2006.06.003 17336792

[bib37] Maloney MJ, McGuire JB, Daniels SR. Reliability testing of a children's version of the Eating Attitude Test. J Am Acad Child Adolesc Psychiatry 1988; 27: 541–543. 3182615 10.1097/00004583-198809000-00004

[bib38] Wong Y, Chang YJ, Tsai MR, Liu TW, Lin W. The body image, weight satisfaction, and eating disorder tendency of school children: the 2-year follow-up study. J Am Coll Nutr 2011; 30: 126–133. 21730220 10.1080/07315724.2011.10719951

[bib39] Follansbee-Junger K, Janicke DM, Sallinen BJ. The influence of a behavioral weight management program on disordered eating attitudes and behaviors in children with overweight. J Am Diet Assoc 2010; 110: 1653–1659. 21034878 10.1016/j.jada.2010.08.005 PMC3038551

[bib40] Colton PA, Olmsted MP, Rodin GM. Eating disturbances in a school population of preteen girls: assessment and screening. Int J Eat Disord 2007; 40: 435–440. 17497707 10.1002/eat.20386

[bib41] Oken E, Levitan EB, Gillman MW. Maternal smoking during pregnancy and child overweight: systematic review and meta-analysis. Int J Obes 2008; 32: 201–210. 10.1038/sj.ijo.0803760 PMC2586944 18278059

[bib42] Davey Smith G, Sterne JAC, Fraser A, Tynelius P, Lawlor DA, Rasmussen F. The association between BMI and mortality using offspring BMI as an indicator of own BMI: large intergenerational mortality study. BMJ 2009; 339: b5043. 20028778 10.1136/bmj.b5043 PMC2797052

[bib43] Angrist JD, Imbens GW, Rubin DB. Identification of causal effects using instrumental variables. J Am Stat Assoc 1996; 91: 444–455.

[bib44] Ranzenhofer LM, Tanofsky-Kraff M, Menzie CM, Gustafson JK, Ruteldge MS, Keil MF et al. Structure analysis of the Children's Eating Attitudes Test in overweight and at-risk for overweight children and adolescents. Eat Behav 2007; 9: 218–227. 18329601 10.1016/j.eatbeh.2007.09.004 PMC2291293

[bib45] Hudson JI, Hiripi E, Pope HGJr, Kessler RC. The prevalence and correlates of eating disorders in the National Comorbitidy Survey Replication. Biol Psychiatry 2007; 61: 348–358. 16815322 10.1016/j.biopsych.2006.03.040 PMC1892232

[bib46] Marchi M, Cohen P. Early-childhood eating behaviours and adolescent eating disorders. J Am Acad Child Adolesc Psychiatry 1990; 29: 112–117. 2295562 10.1097/00004583-199001000-00017

[bib47] Field AE, Javaras KM, Aneja P, Kitos N, Camargo CA, Taylor CB et al. Family, peer and media predictors of becoming eating disordered. Arch Pediatr Adol Med 2008; 162: 574–579. 10.1001/archpedi.162.6.574 PMC3652375 18524749

[bib48] Wardle J, Carnell S, Haworth CM, Plomin R. Evidence for a strong genetic influence on childhood adiposity despite the force of the obesogenic environment. Am J Clin Nutr 2008; 87: 398–404. 18258631 10.1093/ajcn/87.2.398

[bib49] Schousboe K, Visscher PM, Erbas B, Kyvik KO, Hopper JL, Henriksen JE et al. Twin study of genetic and environmental influences on adult body size, shape and composition. Int J Obes 2003; 28: 39–48. 10.1038/sj.ijo.0802524 14610529

[bib50] Danielzik S, Czerwinski-Mast M, Langnäse K, Dilba B, Müller MJ. Parental overweight, socioeconomic status and high birth weight are the major determinants of overweight and obesity in 5-7 y-old children: baseline data of the Kiel Obesity Prevention Study (KOPS). Int J Obes 2004; 28: 1494–1502. 10.1038/sj.ijo.0802756 15326465

[bib51] Smolak L, Thompson JK. Body Image Eating Disorders and Obesity in Youth: Assessment Prevention, and Treatment. American Psychological Association: Washington, DC, 2002.

[bib52] Go AS, Mozaffarian D, Roger VL, Benjamin EJ, Berry JD, Borden WB et al. Overweight and obesity in heart disease and stroke statistics-2013 update: a report from the American Heart Association. Circulation 2013; 127: e6–e245. 23239837 10.1161/CIR.0b013e31828124ad PMC5408511

[bib53] Klump KL, McGue M, Iacono WG. Differential heritability of eating attitudes and behaviours in prepubertal versus pubertal twins. Int J Eat Disord 2003; 33: 287–292. 12655625 10.1002/eat.10151

[bib54] Kotler LA, Cohen P, Davies M, Pine DS, Walsh BT. Longitudinal relationships between childhood, adolescent, and adult eating disorders. J Am Acad Child Adolesc Psychiatry 2001; 40: 1434–1440. 11765289 10.1097/00004583-200112000-00014

[bib55] Maloney MJ, McGuire J, Daniels SR, Specker B. Dieting behaviour and eating attitudes in children. Pediatrics 1989; 84: 482–489. 2788865

[bib56] Sancho C, Asorey O, Arija V, Canals J. Psychometric characteristics of the Children's Eating Attitudes Test in a Spanish sample. Eur Eat Disord Rev 2005; 13: 338–343.

