# Supplementary material for: Prospective associations of parental smoking, alcohol use, marital status, maternal satisfaction, and parental and childhood body mass index at 6.5 years with later problematic eating attitudes
Source: Nutr Diabetes. 2014 Jan 6;4(1):e100–. doi: 10.1038/nutd.2013.40 (PMC3904081; doi:10.1038/nutd.2013.40)
Supplement: Supplementary Information [file nutd201340x1.doc]

**Supplementary Information**

Please find attached the supplementary information for the manuscript entitled “Prospective associations of parental smoking, alcohol use, marital status, maternal satisfaction, and parental and childhood body mass index at 6.5 years with later problematic eating attitudes”.

1. **Supplementary Table 1:** Factor analysis showing performance of ChEAT-24 questionnaire in PROBIT, Belarus
2. **Supplementary Table 2:** Sensitivity analysis of associations between parental and child BMI and ChEAT scores ≥85^th^ percentile excluding polyclinic site “L” in PROBIT, Belarus
3. **Supplementary Table 3:** Sensitivity analysis of associations between parental and child BMI and ChEAT scores ≥91^st^ percentile (including polyclinic site “L”) in PROBIT, Belarus
4. **Supplementary Table 4:** Instrumental variable analysis using maternal and paternal BMI as instruments for child BMI in PROBIT, Belarus
5. **Supplementary Figure 1:** CONSORT flow diagram of progress of clusters and individuals through PROBIT recruitment and follow-up phases 1, II and III in PROBIT, Belarus

**Supplementary Table 1: Factor analysis showing performance of ChEAT-24 questionnaire in PROBIT, Belarus**

| Item Number | Question | Loading |
| --- | --- | --- |
| **Factor 1 (18.97% variance explained): weight preoccupation** | | |
| 1 | Scared about overweight | 0.73 |
| 10 | Feel guilty after eating | 0.40 |
| 11 | Think about being thinner | 0.82 |
| 12 | Exercise to burn energy | 0.50 |
| 14 | Think about body fat | 0.79 |
| 23 | I have been dieting | 0.37 |
|  |  |  |
| **Factor 2 (10.61% variance explained): pressure from others** | | |
| 8 | Others want me to eat more | 0.85 |
| 13 | Others think I’m too thin | 0.78 |
| 15 | Take longer than others to eat | 0.34 |
| 20 | Others pressure me to eat | 0.86 |
|  |  |  |
| **Factor 3 (6.20% variance explained): restriction and control** | | |
| 2 | Stay away from eating when hungry | 0.69 |
| 6 | Aware of calorie content | 0.37 |
| 7 | Stay away from carbohydrates | 0.68 |
| 10 | Feel guilty after eating | 0.31 |
| 16 | Stay away from sugars | 0.58 |
| 24 | Like stomach to be empty | 0.57 |
|  |  |  |
| **Factor 4 (5.82% variance explained): food preoccupation** | | |
| 3 | Think about food a lot | 0.74 |
| 4 | Have gone on eating binges | 0.59 |
| 15 | Take longer than others to eat | 0.33 |
| 21 | Too much thought into food | 0.78 |
|  |  |  |
| **Factor 5 (5.60% variance explained): dieting** | | |
| 5 | Cut food into small pieces | 0.51 |
| 6 | Aware of calorie content | 0.49 |
| 12 | Exercise to burn energy | 0.36 |
| 16 | Stay away from sugars | 0.39 |
| 17 | Eat diet foods | 0.59 |
| 18 | Food controls my life | 0.53 |
| 22 | Uncomfortable after eating sweets | 0.31 |

**Supplementary Table 2: Sensitivity analysis of associations between parental and child BMI and ChEAT scores ≥85^th^ percentile excluding polyclinic site “L” in PROBIT, Belarus**

| **Exposures^1^** | **Percentage of ChEAT scores ≥ 22.5** | **Basic Model^2^** | **Fully Adjusted Model^3^** |
| --- | --- | --- | --- |
| **Child’s BMI at PROBIT II (kg/m^2^)** |  |  |  |
| Normal (n = 10 817) | 17.1 | 1.00 (ref) | 1.00 (ref) |
| Underweight (n = 284) | 15.9 | 0.87 (0.62, 1.22) | 0.85 (0.59, 1.20) |
| Overweight (n = 929) | 30.3 | 2.18 (1.86, 2.55) | 2.15 (1.82, 2.53) |
| Obese (n = 260) | 41.9 | 3.76 (2.87, 4.94) | 3.84 (2.90, 5.09) |
| OR (95% CI) per SD increase in BMI |  | 1.33 (1.28, 1.39) | 1.34 (1.28, 1.40) |
| *P*-value for trend |  | <0.0001 | <0.0001 |
|  |  |  |  |
| **Maternal BMI at PROBIT II (kg/m^2^)** |  |  |  |
| Normal (n = 7 557) | 17.9 | 1.00 (ref) | 1.00 (ref) |
| Underweight (n = 71) | 18.3 | 0.98 (0.53, 1.83) | 1.19 (0.63, 2.24) |
| Overweight (n = 3 147) | 18.9 | 1.10 (0.98, 1.23) | 1.10 (0.98, 1.23) |
| Obese (n = 1 364) | 21.6 | 1.29 (1.11, 1.50) | 1.30 (1.11, 1.51) |
| OR (95% CI) per SD increase in BMI |  | 1.10 (1.05, 1.15) | 1.10 (1.05, 1.16) |
| *P*-value for trend |  | 0.0001 | 0.0002 |
|  |  |  |  |
| **Paternal BMI at PROBIT II (kg/m^2^)** |  |  |  |
| Normal (n = 5 240) | 17.8 | 1.00 (ref) | 1.00 (ref) |
| Overweight (n = 4 779) | 19.1 | 1.09 (0.98, 1.21) | 1.11 (0.99, 1.23) |
| Obese (n = 1 063) | 21.7 | 1.29 (1.09, 1.53) | 1.28 (1.07, 1.52) |
| OR (95% CI) per SD increase in BMI |  | 1.09 (1.03, 1.14) | 1.09 (1.03, 1.14) |
| *P*-value for trend |  | 0.001 | 0.001 |

*BMI = Body mass index (kg/m^2^); ChEAT = Children’s Eating Attitudes Test (35); CI = confidence interval; OR = Odds Ratio; PROBIT = Promotion of Breastfeeding Intervention Trial; ref = reference; SD = Standard Deviation.*

*^1^Categories of BMI for underweight, overweight and obesity in children were defined by Cole et al. and are mapped onto the WHO categories for adults (32, 33). The WHO definitions were used for adults.*

*^2^All effect-estimates are adjusted for age, sex and clustering by hospital/polyclinic and represent the odds ratio (OR) giving the change in odds (95% CI) of having a ChEAT score ≥22.5 (85th percentile) per SD increase in BMI (kg/m^2^).*

*^3^Adjusted for age, sex, strata, treatment group, maternal/paternal occupation/education, maternal smoking status from pregnancy to PROBIT II and polyclinic site (polyclinic site “L” excluded).*

**Supplementary Table 3: Sensitivity analysis of associations between parental and child BMI and ChEAT scores ≥91^st^ percentile in PROBIT, Belarus**

| **Exposures^1^** | **Percentage of ChEAT scores ≥ 25.5** | **Basic Model^2^** | **Fully Adjusted Model^3^** |
| --- | --- | --- | --- |
| **Child’s BMI at PROBIT II (kg/m^2^)** |  |  |  |
| Normal (n = 11 293) | 10.2 | 1.00 (ref) | 1.00 (ref) |
| Underweight (n = 290) | 7.9 | 0.70 (0.45, 1.09) | 0.68 (0.43, 1.08) |
| Overweight (n = 993) | 18.2 | 2.03 (1.69, 2.43) | 2.09 (1.73, 2.52) |
| Obese (n = 275) | 30.9 | 4.18 (3.15, 5.55) | 4.38 (3.27, 5.86) |
| OR (95% CI) per SD increase in BMI |  | 1.35 (1.29, 1.42) | 1.37 (1.31, 1.44) |
| *P*-value for trend |  | <0.0001 | <0.0001 |
|  |  |  |  |
| **Maternal BMI at PROBIT II (kg/m^2^)** |  |  |  |
| Normal (n = 7 949) | 10.6 | 1.00 (ref) | 1.00 (ref) |
| Underweight (n = 75) | 10.7 | 0.97 (0.46, 2.07) | 1.13 (0.53, 2.44) |
| Overweight (n = 3 265) | 11.5 | 1.10 (0.97, 1.26) | 1.10 (0.95, 1.26) |
| Obese (n = 1 430) | 14.0 | 1.39 (1.17, 1.65) | 1.37 (1.14, 1.63) |
| OR (95% CI) per SD increase in BMI |  | 1.11 (1.05, 1.17) | 1.11 (1.05, 1.17) |
| *P*-value for trend |  | 0.0003 | 0.0005 |
|  |  |  |  |
| **Paternal BMI at PROBIT II (kg/m^2^)** |  |  |  |
| Normal (n = 5 461) | 10.6 | 1.00 (ref) | 1.00 (ref) |
| Overweight (n = 5 010) | 11.3 | 1.09 (0.96, 1.24) | 1.09 (0.96, 1.25) |
| Obese (n = 1 133) | 13.1 | 1.30 (1.07, 1.59) | 1.32 (1.07, 1.63) |
| OR (95% CI) per SD increase in BMI |  | 1.10 (1.04, 1.16) | 1.10 (1.04, 1.17) |
| *P*-value for trend |  | 0.001 | 0.001 |

*BMI = Body mass index (kg/m^2^); ChEAT = Children’s Eating Attitudes Test (35); CI = confidence interval; OR = Odds Ratio; PROBIT = Promotion of Breastfeeding Intervention Trial; ref = reference; SD = Standard Deviation.*

*^1^Categories of BMI for underweight, overweight and obesity in children were defined by Cole et al. and are mapped onto the WHO categories for adults (32, 33). The WHO definitions were used for adults.*

*^2^All effect-estimates are adjusted for age, sex and clustering by hospital/polyclinic and represent the odds ratio (OR) giving the change in odds (95% CI) of having a ChEAT score ≥25.5 (91th percentile) per SD increase in BMI (kg/m^2^).*

*^3^Adjusted for age, sex, strata, treatment group, maternal/paternal occupation/education, maternal smoking status from pregnancy to PROBIT II and polyclinic site.*

**Supplementary Table 4: Instrumental variable analysis using maternal and paternal BMI as instruments for child BMI in PROBIT, Belarus**

|  | **Odds Ratios (95% CI) of ChEAT score ≥85^th^ percentile per SD increase in child’s BMI (kg/m^2^)^1^** | | |
| --- | --- | --- | --- |
| **Exposure** | **Estimate using child BMI entered in a conventional linear regression analysis** | **Instrumental variable estimate of the effect of child BMI using maternal BMI as an instrument** | **Instrumental variable estimate of the effect of child BMI using paternal BMI as an instrument** |
| **BMI Z-score** | 1.34 (1.29, 1.40),  *P*<0.0001 | 1.46 (1.28, 1.67),  *P*<0.0001 | 1.42 (1.22, 1.65)  *P*<0.0001 |

*BMI = Body mass index (kg/m^2^); ChEAT = Children’s Eating Attitudes Test (35); CI = confidence interval; PROBIT = Promotion of Breastfeeding Intervention Trial; SD = Standard deviation.*

*^1^Odds ratios* *adjusted for age, sex and clustering by hospital/polyclinic and represent the odds ratio (OR) giving the change in odds (95% CI) of having a ChEAT score ≥22.5 (85^th^ percentile) per SD increase in child BMI (kg/m^2^) at PROBIT II.*

**Supplementary Figure 1: Flow diagram of progress of clusters and individuals through PROBIT recruitment and follow-up phases I, II and III in PROBIT, Belarus**

| Enrolment | 34 maternity hospitals and associated polyclinics assessed for eligibility and pair-matched | | | | | | | | | | | | | | | | | | | | | | | | | | | | | | |
| --- | --- | --- | --- | --- | --- | --- | --- | --- | --- | --- | --- | --- | --- | --- | --- | --- | --- | --- | --- | --- | --- | --- | --- | --- | --- | --- | --- | --- | --- | --- | --- |
|  |  |  |  |  |  |  |  |  |  |  |  |  |  |  |  |  |  |  |  |  |  |  |  |  |  |  |  |  |  |  |  |
| Allocation | 17 pairs cluster randomized | | | | | | | | | | | | | | | | | | | | | | | | | | | | | | |
|  |  |  |  |  |  |  |  |  |  |  |  |  |  |  |  |  |  |  |  |  |  |  |  |  |  |  |  |  |  |  |  |
|  |  |  |  |  |  |  |  |  |  |  |  |  |  |  |  |  |  |  |  |  |  |  |  |  |  |  |  |  |  |  |  |
|  | 17 sites allocated to experimental breastfeeding intervention:  16 sites implemented the allocated intervention  1 site declined to participate and did not receive allocated intervention | | | | | | | | | | | | | | |  | | | 17 sites allocated to standard care (control):  6 sites continued standard care  1 site declined to participate and did not receive allocated control | | | | | | | | | | | | |
|  |  | | | | | |  | | | | | | | |  | | |  | | | |  |  | | | | | | | | |
|  |  | | | | | |  | | | | | | | |  | | |  | | | |  | 1 site excluded (falsified outcome data, 749 mother-infant pairs excluded) | | | | | | | | |
|  |  |  |  |  |  |  |  |  |  |  |  |  |  |  |  |  |  |  |  |  |  |  |  |  |  |  |  |  |  |  |  |
|  |  | | | | | |  | | | | | | | |  | | |  | | | |  | | | | | | | | | |
| Recruitment | 8 865 mother-infant pairs recruited to intervention sites (16 sites, median cluster size=501, range=249-1180) | | | | | | | | | | | | | | |  | | | 8 181 mother-infant pairs recruited to control sites (15 sites, median cluster size=461, range=232-940) | | | | | | | | | | | | |
|  |  | | | | |  | | | | | | | | |  | | |  | | |  | | | | | | | | | |  |
| Follow-up PROBIT I  at 12m | No sites lost to follow-up | | | | | | | | |  | | | | | |  | | | No sites lost to follow-up | | | | | | | |  | | | | |
|  | 8 569 mother-infant pairs attended^1^ | | | | 276  mother-infant pairs did not attend | | | | |  | 20 infants died before 12 months | | | | |  | | | 7 923 mother-infant pairs attended^1^ | | 230 mother-infant pairs did not attend | | | | | |  | | 28 infants died before 12 months | | |
|  |  |  |  |  |  |  |  |  |  |  |  |  |  |  |  |  |  |  |  |  |  |  |  |  |  |  |  |  |  |  |  |
|  |  |  | | | | | |  | | | | | | | |  | | |  |  | | | |  | | | | | | | |
|  |  | | | | |  | | | | | | | | | |  | | |  | |  | | | | | | | | | | |
| Follow-up PROBIT II  at 6.5 years | No sites lost to follow-up | | | | | | | | |  | | | | | |  | | | No sites lost to follow-up | | | | | | | |  | | | | |
|  | 7 108 children attended | | | | 1 717 children did not attend | | | | |  | 20 children died | | | | |  | | | 6 781 children attended | | 1 354 children did not attend | | | | | |  | | 18 children died | | |
|  |  |  |  |  |  |  |  |  |  |  |  |  |  |  |  |  |  |  |  |  |  |  |  |  |  |  |  |  |  |  |  |
|  |  |  | | | | | |  | | | | | | | |  | | |  |  | | | |  | | | | | | | |
|  |  | | |  | | | | | | | | | | | |  | | |  | |  | | | | | | | | | | |
| Follow-up PROBIT III  at 11.5 years | No sites lost to follow-up | | | | | | | | |  | | | | | |  | | | No sites lost to follow-up | | | | | | |  | | | | | |
|  | 7 405 children (16 sites, median cluster size=385, range=155-1027) | | | | 1 460 children excluded:  1 414 did not attend  46 died in total | | | | |  | 6 children died | | | | |  | | | 6 474 children (15 site, median cluster size=318, range=160-857) | | 1 707 children excluded:  1 656 did not attend  51 died in total | | | | |  | | 5 children died | | | |
|  |  |  |  |  |  |  |  |  |  |  |  |  |  |  |  |  |  |  |  |  |  |  |  |  |  |  |  |  |  |  |  |
|  |  |  |  |  |  |  |  |  |  |  |  |  |  |  |  |  |  |  |  |  |  |  |  |  |  |  |  |  |  |  |  |
|  | | |  | | | | |  |  | | | |  | | | | | | |  | | | | |  | | | | |  | |
|  | | | | | |  | | | | | |  | |  | | | | | | |  | | | | | | | | |  | |

| 7 352 children with completed ChEAT data |  | 6 399 children with completed ChEAT data |
| --- | --- | --- |

*ChEAT = Children’s Eating Attitudes Test (35); PROBIT = Promotion of Breastfeeding Intervention Trial.*

*^1^The numbers of mother-infant pairs with 12 months completed follow-up, and the number of infants who had died before 12 months, differ slightly compared to those originally reported in reference 15 because of continued work on retrieving previously unreturned data forms from the polyclinics and continued data cleaning (Intervention: 8 569 pairs vs. 8 547 originally reported; control: 7 923 pairs vs. 7 895 originally reported; 48 died vs 49 originally reported).*
